# Genomic and neural analysis of the estradiol-synthetic pathway in the zebra finch

**DOI:** 10.1186/1471-2202-11-46

**Published:** 2010-04-01

**Authors:** Sarah E London, David F Clayton

**Affiliations:** 1 Institute for Genomic Biology, University of Illinois at Urbana-Champaign, Urbana, IL, USA; 2 Beckman Institute of Advanced Science and Technology, University of Illinois at Urbana-Champaign, Urbana, IL, USA; 3 Department of Cell and Developmental Biology, University of Illinois at Urbana-Champaign, Urbana, IL, USA

## Abstract

**Background:**

Steroids are small molecule hormones derived from cholesterol. Steroids affect many tissues, including the brain. In the zebra finch, estrogenic steroids are particularly interesting because they masculinize the neural circuit that controls singing and their synthesis in the brain is modulated by experience. Here, we analyzed the zebra finch genome assembly to assess the content, conservation, and organization of genes that code for components of the estrogen-synthetic pathway and steroid nuclear receptors. Based on these analyses, we also investigated neural expression of a cholesterol transport protein gene in the context of song neurobiology.

**Results:**

We present sequence-based analysis of twenty steroid-related genes using the genome assembly and other resources. Generally, zebra finch genes showed high homology to genes in other species. The diversity of steroidogenic enzymes and receptors may be lower in songbirds than in mammals; we were unable to identify all known mammalian isoforms of the 3β-hydroxysteroid dehydrogenase and 17β-hydroxysteroid dehydrogenase families in the zebra finch genome assembly, and not all splice sites described in mammals were identified in the corresponding zebra finch genes. We did identify two factors, Nobox and NR1H2-RXR, that may be important for coordinated transcription of multiple steroid-related genes. We found very little qualitative overlap in predicted transcription factor binding sites in the genes for two cholesterol transport proteins, the 18 kDa cholesterol transport protein (TSPO) and steroidogenic acute regulatory protein (StAR). We therefore performed in situ hybridization for TSPO and found that its mRNA was not always detected in brain regions where StAR and steroidogenic enzymes were previously shown to be expressed. Also, transcription of TSPO, but not StAR, may be regulated by the experience of hearing song.

**Conclusions:**

The genes required for estradiol synthesis and action are represented in the zebra finch genome assembly, though the complement of steroidogenic genes may be smaller in birds than in mammals. Coordinated transcription of multiple steroidogenic genes is possible, but results were inconsistent with the hypothesis that StAR and TSPO mRNAs are co-regulated. Integration of genomic and neuroanatomical analyses will continue to provide insights into the evolution and function of steroidogenesis in the songbird brain.

## Background

Steroids are central to zebra finch (*Taeniopygia guttata*) neurobiology. They are essential for early developmental organization of the song control system, and they continue to modulate brain and behavior throughout life [1,2]. Although some steroids are supplied to the brain from the periphery, others including estradiol can be synthesized within the brain, either *de novo *from cholesterol or by metabolism of precursor steroids that originate in the periphery, as shown by evidence from biochemical enzyme activity assays, explant and dissociated culture analysis, molecular identification and neuroanatomical mapping of steroidogenic factors, and *in vivo *steroid measurements [1,3-19] London, Itoh, Lance, Ekanayake, Oyama, Arnold, Schlinger: Neural expression and post-transcriptional dosage compensation of the steroid metabolic enzyme 17β-HSD type 4: submitted. Steroids synthesized within the brain, termed "neurosteroids," masculinize the song system and can be rapidly modulated by experience [1,5,8]. Steroids often act through nuclear receptor transcription factors, which can be abundant in the songbird brain, including in the song control system [20-23]. Thus the zebra finch brain has the capacity to both produce and respond to a variety of steroids that can alter neural function and song behavior.

**Figure 1:**
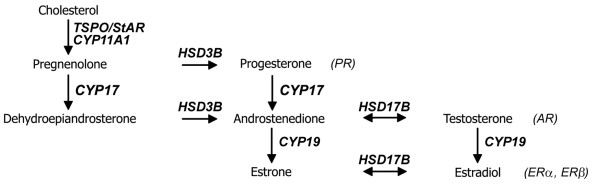
**The estradiol-synthetic pathway and nuclear receptors**. Cholesterol is the universal steroid substrate. Initiation of steroidogenesis begins with the transport of cholesterol, via the action of the 18 kDa cholesterol transport protein (TSPO) and/or the steroidogenic acute regulatory protein (StAR), to the first enzyme in the pathway, cytochrome P450 side chain cleavage (CYP11A1). From pregnenolone, four more enzymes are required to produce estradiol: 3β-hydroxsteroid dehydrogenase (HSD3B), cytochrome P450 17α-hydroxylase/17,20 lyase (CYP17), 17β-hydroxysteroid dehydrogenases (HSD17B), and cytochrome P450 aromatase (CYP19). Cholesterol transport proteins and enzymes are in bold italics. Steroids are in plain text. The four major nuclear receptors for the three classes of steroids produced along the estradiol-synthetic pathway are in italicized parentheses: progesterone receptor (PR), estrogen receptor α (ERα), estrogen receptor β (ERβ), and androgen receptor (AR).

Steroids are small molecule hormones derived from cholesterol through a series of enzymatic conversions (Figure 1). The first steroidogenic enzyme resides in the inner mitochondrial membrane, and the rate-limiting step of steroidogenesis is the transport of cholesterol across the outer mitochondrial membrane. Two major cholesterol transport proteins, which have been proposed to work in concert as part of a protein complex, are the steroidogenic acute regulatory protein (StAR) and the 18 kDa cholesterol transport protein (TSPO; previously named peripheral type benzodiazepine receptor) [24-29]. Steroid synthesis starts with the action of cytochrome P450 side chain cleavage (CYP11A1), which produces pregnenolone. Pregnenolone can be converted to either progesterone or dehydroepiandrosterone via the action of cytochrome P450 17α-hydroxylase/17,20 lyase (CYP17) or 3β-hydroxsteroid dehydrogenase/Δ5,Δ4 isomerase (HSD3B1), respectively. Androstenedione is produced from progesterone through the activity of HSD3B1 and from dehydroepiandrosterone through the activity of CYP17. Androstenedione can be converted to testosterone or an estrogen, estrone, via the activity of 17β-hydroxysteroid dehydrogenases (HSD17B) or cytochrome P450 aromatase (CYP19), respectively. HSD17B can also convert estrone to estradiol, and CYP19 metabolizes testosterone into estradiol. Of note is the fact that multiple HSD3B and HSD17B types exist in other animals, and that several of HSD17B enzymes can use androgens and estrogens as substrates [30-32].

Steroids most commonly act by binding to nuclear receptors that dimerize, translocate to the nucleus, and function as DNA-binding transcription factors. The steroid receptors belong to nuclear receptor class 3, and maintain the typical four-domain structure of receptors in the nuclear receptor superfamily [33]. There are four main nuclear receptors for the steroids produced along the estradiol-synthetic pathway: estrogens bind estrogen receptor alpha (ERα) and beta (ERβ); androgens bind androgen receptor (AR), and progesterone binds progestin receptors (PR).

With the assembly of the zebra finch genome sequence, we are now in position for the first time to assess the organization and regulation of the network of genes that control steroid synthesis and function in the songbird brain. Starting with the set of Ensembl gene predictions for the zebra finch, we manually curated the assembled models for genes that encode the major components of the estradiol-synthetic pathway and related proteins, as well as four nuclear steroid receptors. We characterized the structure, diversity, and evolutionary conservation of these genes. This analysis led us to examine the neural expression patterns of a cholesterol transport protein in the context of song neurobiology.

## Results

### Characterization of steroidogenic genes in the zebra finch genome assembly

We used all available resources (including Ensembl gene models, alignments to brain expressed sequence tags (ESTs) and GenBank cDNA clone entries, and cross-species and functional domain homology searches) to identify the sequences of twenty steroid-related genes in the zebra finch genome assembly (Table 1). Of these, two genes code for cholesterol transport proteins (StAR and TSPO), five genes code for enzymes known to be active in the estradiol-synthetic pathway in songbirds (CYP11A1, HSD3B1, CYP17, CYP19, HSD17B4), nine genes code for related HSD3B and HSD17B enzymes, some of which are likely to also be involved in the estrogen-synthetic pathway (HSD3B7, HSD17B1, HSD17B2, HSD17B3, HSD17B6, HSD17B7, HSD17B10, HSD17B11, HSD17B12), and four genes code for the primary nuclear receptors for the steroids produced in the estradiol-synthetic pathway (ERα, ERβ, AR, and PR).

**Table 1:** Summary of steroid-related genes identified in the zebra finch genome assembly.

	Ensembl model ID	Chromosomal location	Alternate location
**StAR**	ENSTGUG00000004778	chr22:2,790,291-2,803,402	
**TSPO**	ENSTGUG00000012033	chr1A:64,600,558-64,604,825	
**CYP11A1**	ENSTGUG00000016385	chrUn:111,323,270-111,327,067	
**HSD3B1**	ENSTGUG00000013351	chr1:90,752,924-90,765,922	
**HSD3B7**	ENSTGUG00000004368	chr19:4700526-4724260	
**CYP17**	ENSTGUG00000010219	chr6:22,934,143-22,936,206	
**HSD17B1**	ENSTGUG00000002682	chr27:2,575,793-2,577,330	
**HSD17B2**	ENSTGUG00000004388	chr11:2,151,325-2,156,358	chrUn:30,861,771-30,869,026
**HSD17B3**		chrZ:9,425,091-9,449,191	
**HSD17B4**	ENSTGUG00000001154	chrZ:24,424,040-24,517,666	
**HSD17B6**		chrUn:44,543,014-44,547,896	
**HSD17B7**	ENSTGUG00000017081	chr8_random:518,775-526,426	
**HSD17B10**	ENSTGUG00000015458	chrUn:14,031,915-14,032,480	
**HSD17B11**	ENSTGUG00000002260	chr4:8,273,553-8,281,927	
**HSD17B12**	ENSTGUG00000010212	chr5:19,883,132-19,948,030	
**CYP19**	ENSTGUG00000006993	chr10:9,056,446-9,074,753	
**promoter 1a**		chr10:9,078,023-9,078,512	
**promoter 1b**		chr10:9,052,768-9,083,661	chrUn:18,857,683-18,860,112
**ERα**	ENSTGUG00000011249	chr3:56,288,003-56,500,000	
**ERβ**	ENSTGUG00000012942	chr5:54,900,184-54,948,467	
**PR**	ENSTGUG00000012778	chr1:77374938-77451302	
**AR**	ENSTGUG00000002760	chr4A: 6,416,086-6,447,982	

Genomic information for all twenty genes examined. Ensembl gene identifiers are listed for the eighteen genes with official models (HSD17B3 and HSD17B6 do not have Ensembl gene models). Chromosomal positions are also listed, as are the locations of either alternative mappings (HSD17B2) or annotated promoter regions (CYP19).

**Figure 2:**
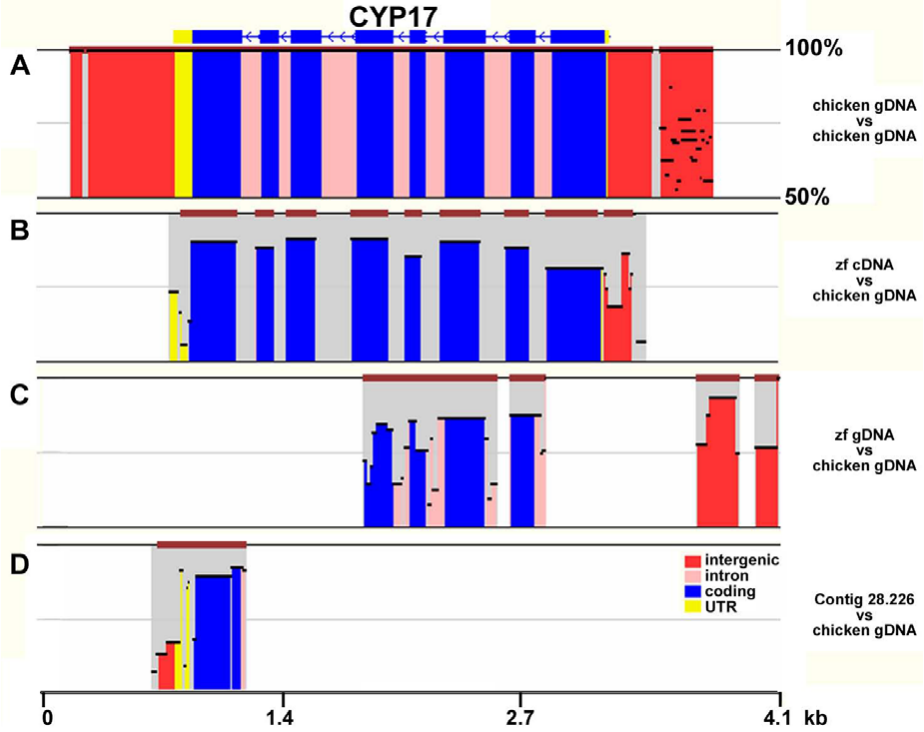
**Alignments of chicken and zebra finch CYP17 sequences**. Homology of chicken CYP17 gene (gDNA) and several zebra finch CYP17 sequences, as depicted in zpicture. A) Comparison of chicken gDNA to itself, to illustrate structure of the gene, B) comparison of a full length zebra finch CYP17 cDNA clone sequence (Accession numbers AY313844 and AY313845) to chicken gDNA, C) comparison of the CYP17 gene sequence obtained from the zebra finch genomic assembly to the chicken gDNA, showing substantial missing sequence, and D) comparison of zebra finch Contig 28.226 sequence, a contig that was not incorporated into the assembly, to the 3' end of the CYP17 gene. Note the scale of homology is from 50-100%, and that arrows in top block denote the chicken gene sequence is oriented so that the 5' end is on the right.

Complete coding regions are present for the four receptor genes and for the HSD3B1, CYP19, HSD17B4, HSD17B6, HSD17B11 genes, but only partial gene sequences were identified for the other genes in the assembly. In cases where full length cDNA clone sequences were available, we could determine that incomplete gene models were due to assembly gaps. Some genome assembly gaps result in very small omissions (e.g. StAR is missing ~100 bp of exon 1), but others are large (e.g. CYP11A1 and CYP17 are missing 5 and 4 exons, respectively). In the case of CYP17, we identified a genome contig in the trace archive (Contig 28.226) that codes for exon 8 and a portion of the 3'UTR that was not included in the current genome assembly (Figure 2). The structures of each of the zebra finch genes are illustrated by their evolutionarily conserved regions when compared to chicken or human genes in zpicture format (http://zpicture.dcode.org/; Additional File 1, Figure S1).

HSD3B is a family with up to seven enzymes whose members each derive from unique genes, almost all of which are located along the same chromosome in human and mouse [31,34-38]. We used homology searches to try to identify zebra finch genes for all of the known HSD3B genes. HSD3B type 1 is annotated in the genome on chromosome 1. Several other types of HSD3B genes from other species show homology to this same location, but BLAT analysis with the zebra finch HSD3B1 cDNA clone sequence aligns only with this one position, suggesting that there is only one HSD3B gene in the zebra finch assembly. Homology searches with the conserved Rossmann catalytic domain did not identify any additional putative HSD3B genes on chromosome 1, further supporting the conclusion that the HSD3B1 gene is the only one on chromosome 1. We did, however, identify an unannotated, incomplete gene for HSD3B type 7 on chromosome 19 by homology searches.

The HSD17B enzymes are also a family of similar enzymes. Fourteen HSD17B genes have been described to date in humans, and multiple HSD17B genes have also been cloned from or identified in the genomes of other mammals, fish, and chicken [32,36,39]. We attempted to find all fourteen genes in the zebra finch genome assembly. We identified genes for HSD17B 1-4, 6, 7, 10-12 in the assembly (numbering follows that of the human genes). We confirmed the available HSD17B1 genomic sequence (below). Two genes, HSD17B3 and HSD17B4, were mapped to the Z sex chromosome in the genome assembly; the HSD17B4 mapping is consistent with experimental data that show that this genes is localized to the Z chromosome [40] London, Itoh, Lance, Ekanayake, Oyama, Arnold, Schlinger: Neural expression and post-transcriptional dosage compensation of the steroid metabolic enzyme 17β-HSD type 4: submitted. The Z chromosome position is of note because sex chromosome genes can be expressed at different levels in males and females and have been proposed to contribute to the sexual differentiation of the zebra finch song system [18,41-45] London, Itoh, Lance, Ekanayake, Oyama, Arnold, Schlinger: Neural expression and post-transcriptional dosage compensation of the steroid metabolic enzyme 17β-HSD type 4: submitted. The HSD17B3 gene model is incomplete; there is one gap in the assembly that may remove several coding exons, but the assembly is uninterrupted for up to 65 kb outside of the boundaries of the gene model. It was therefore possible that more gene coding sequence could be obtained through manual curation of this stretch of the assembly. Our attempts to identify additional exons through homology searches were, however, unsuccessful. The HSD17B4 gene shares exon structure with mammalian and chicken HSD17B4 genes and contains only intronic assembly gaps; HSD17B4 was examined in more detail elsewhere [London, Itoh, Lance, Ekanayake, Oyama, Arnold, Schlinger: Neural expression and post-transcriptional dosage compensation of the steroid metabolic enzyme 17β-HSD type 4: submitted].

Several steroidogenic genes have the potential to form alternatively spliced transcripts or utilize alternate promoters. StAR, TSPO, CYP11A1, ERα, ERβ, and AR are predicted to have alternative splice forms in other animals. We identified splice sites in the zebra finch StAR and TSPO genes that give rise to different length transcripts ([46]; Additional File 2, Figure S2). A model for CYP11A1 was not completed because of the large gap in genomic sequence, and we were unable to identify splice variants similar to those in mammals in the zebra finch ERα, ERβ, and AR genes. The first exon of the CYP19 gene is untranslated and contains promoter elements that regulate tissue-specific transcription e.g. [47-53]. In zebra finches as in mammals, there are several variants of exon 1; zebra finch brain CYP19 transcripts almost always use the "1a" exon [48]. Though not annotated as such, we identified the exon 1a sequence in the zebra finch genome assembly approximately 7 kb upstream of the first coding exon (Additional File 2, Figure S2).

### PCR confirmation of genomic sequence

The Ensembl annotation of the genome assembly contains a predicted gene model for HSD17B1. As way of validating the genomic assembly sequence one of the genes that had not been previously cloned, we used the gene model to design PCR primers and successfully amplified the predicted HSD17B1 sequence from zebra finch genomic DNA (Additional File 3, Figure S3). However, we were still unable to obtain the sequence at the 3' end of the gene that is absent in the assembly due to a gap between contigs.

### Phylogeny and selection

Cross-species multiple sequence alignments for each of the twenty genes demonstrated that functional domains were the most highly conserved regions of the genes and that the overall exon/intron structure of these genes is conserved across the fish, bird, and mammal species investigated (see Methods for species; nucleotide alignments not shown, genomic structural homology to chicken or human genes shown in Additional File 1, Figure S1). To examine the relationship between genes in the three families examined here, HSD3B, HSD17B, and the nuclear receptors, we constructed unrooted phylogenetic trees of each of these groups, using predicted protein sequences.

**Figure 3:**
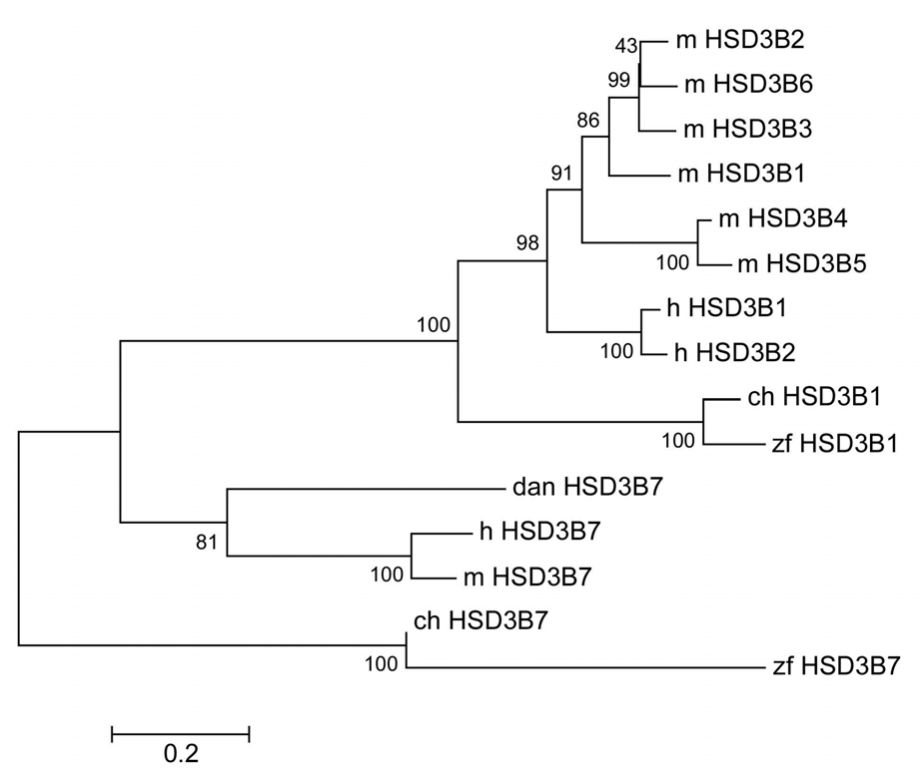
**Unrooted phylogenetic tree of HSD3B predicted protein sequences**. The two HSD3B genes identified in the zebra finch genome assembly, HSD3B1 and HSD3B7, show the closest similarity to the same HSD3B types in the chicken. The HSD3B1 protein sequence is predicted to be more similar to the HSD3B1-6 mammalian proteins than the HSD3B7 zebra finch protein is to the mammalian HSD3B7 protein. Bootstrap values are at branch points. Scale bar denotes substitution rate. zf = zebra finch, ch = chicken, h = human, m = mouse, dan = zebrafish.

Phylogenetic analysis of mouse, human, zebrafish, chicken and zebra finch HSD3B proteins showed that the zebra finch HSD3B1 sequence is most similar to that of the chicken, and groups with the HSD3B1-6 protein sequences of the mouse and human (Figure 3). The HSD3B7 zebra finch and chicken sequences are more similar to HSD3B7 proteins in the other species, but showed less homology compared to human than even the zebrafish sequence.

**Figure 4:**
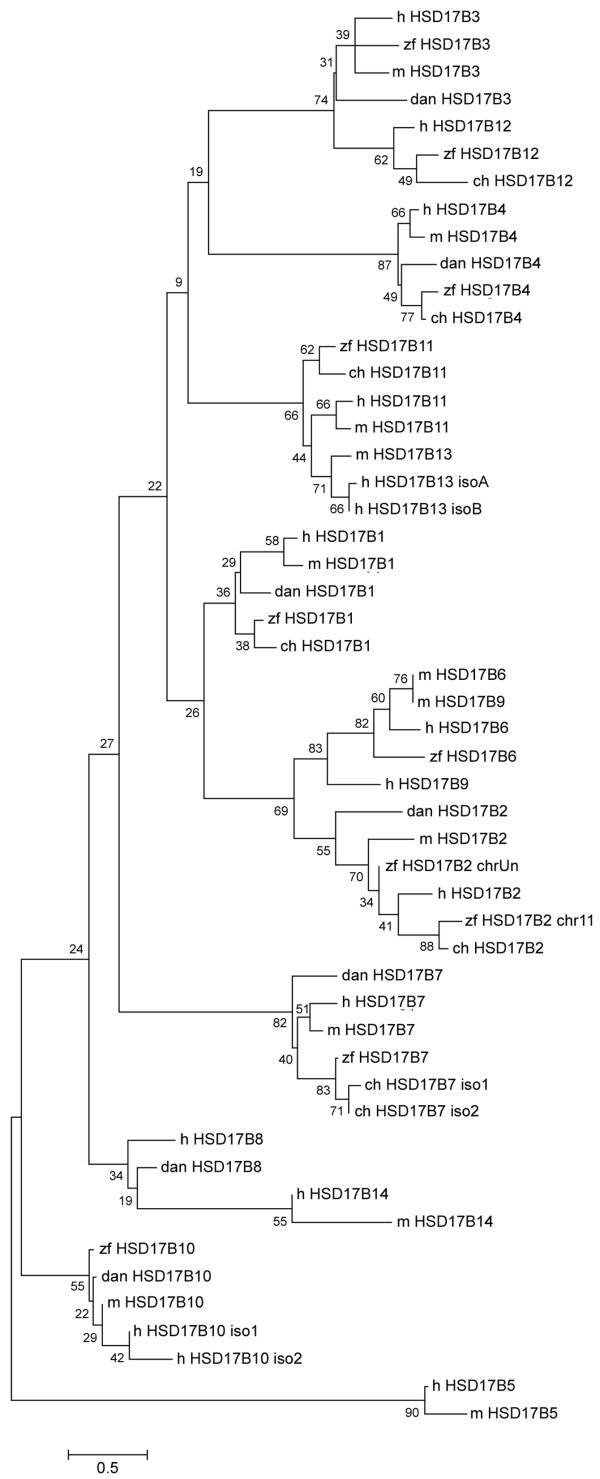
**Unrooted phylogenetic tree of HSD17B predicted protein sequences**. We identified nine HSD17B genes in the zebra finch assembly; these predicted proteins segregated with the same enzyme type in chicken and the other species. The unrooted tree models some enzymes (HSD17B4 and 7) as unique branches. Enzyme types predicted to be evolutionarily related (HSD17B3 and 12, and HSD17B2 and 6), are shown to be preserved in the zebra finch. Bootstrap support values are at branch points. Scale bar denotes substitution rate. zf = zebra finch, ch = chicken, h = human, m = mouse, dan = zebrafish, iso = isoform.

To look at the phylogenetic relationship among HSD17B predicted proteins, we used all annotated HSD17B genes in human, mouse, zebrafish, and chicken, plus those we identified in zebra finch (Figure 4). In general, the zebra finch proteins segregated with the same enzyme type in chicken and the other species. This analysis modeled HSD17B4 and HSD17B7 proteins as independent branches, not closely related to any other HSD17B enzyme. Several other protein types, however, showed more similarity to another. For example, the type 3 and 12 proteins were clustered together on the tree, as were types 2 and 6.

We also examined the nuclear receptors (Additional File 4, Figure S4). The two types of ER segregated together, with the zebra finch ERα and ERβ showing more similarity to their respective orthologs in other species than to each other. Similarly, the AR and PR predicted protein sequences were more similar to each other than to the ERs. For all four types of receptors, the zebra finch is most closely related to the chicken sequence. The zebrafish is the most distantly related to the mammalian proteins, and birds in some cases (ERβ and PR) show more similarity to the mammalian proteins than does the platypus.

### Distribution of predicted transcription factor binding sites

Multiple steroidogenic enzymes need to be present in close proximity to locally synthesize a variety of steroids, including estradiol. The functional connection between these enzymes suggests the possibility that common transcriptional regulatory elements may be shared among the steroidogenic genes to direct their coordinated expression. To pursue this hypothesis, we used a set of Position Weight Matrix (PWM) predictions for specific transcription factor binding sites generated for the whole zebra finch genome as part of the primary analysis of the assembly [54]. We then tested whether any PWM sites were more abundant in territories surrounding steroid related genes than in the genome as a whole. When we used all 18 available Ensembl models, one PWM, NR1H2-RXR, was identified as significantly (p = 0.045) overrepresented in the gene set with the SWAN algorithm [54,55]. No significantly overrepresented PWM sites were identified when subsets of the cholesterol transport and enzymes Ensembl models were used. However, when the four nuclear receptor genes were examined, Nobox was shown to be significantly (p = 0.048) overrepresented according to the SLLR algorithm [55].

Two pairs of functionally related genes - StAR and TSPO, and CYP19 and HSD17B4 - had complete coding regions in the assembly, and few intronic gaps. We therefore inventoried and compared the PWM sites predicted to be in these genes to assess the potential for transcriptional co-regulation (Tables 2 and 3). Comparison of the PWM profiles in StAR and TSPO showed that no sites were common to both genes in the 5'-focused region, and only 23% and 33% of them were shared when the whole gene was taken into account (7/30 for StAR, 7/21 for TSPO). Similarly, when PWM sites for CYP19 and HSD17B4 were compared, only one site was shared in the 5'-focused region, while 89% and 61% (50/56 for CYP19, 50/81 for HSD17B4) were shared when the whole gene was considered.

**Table 2:** Qualitative listing of PWM predictions compiled from StAR and TSPO genes.

	StAR	TSPO	StAR	TSPO		StAR	TSPO	StAR	TSPO
**Ar**					**NFYA**				
**Arnt**					**NHLH1**			□	
**Arnt-Ahr**	■		□		**Nkx2-5**				
**Bapx1**		■		□	**NKX3-1**		■		□
**cEBP**		■		□	**Nobox**			□	
**CREB1**			□		**NR1H2-RXR**				
**Ddit3-Cebpa**		■	□	□	**NR2F1**				
**E2F1**					**NR3C1**		■		□
**ELF5**					**Pax2**				
**ELK1**					**Pax4**				
**En1**					**Pax5**			□	
**ESR1**			□	□	**Pax6**			□	
**ETS1**	■			□	**Pbx**				
**Evi1**					**Pdx1**			□	
**Fos**				□	**PPARG**				□
**Foxa2**					**PPARG-RXRA**				
**FOXC1**					**Prrx2**			□	
**Foxd3**					**REL**				
**FOXF2**		■		□	**RELA**			□	□
**FOXI1**					**REST**			□	□
**FOXL1**					**Roaz**	■		□	
**Foxq1**					**RORA_1**				
**GABPA**			□		**RORA_2**				
**Gata1**					**RORA1**				
**GATA2**					**RREB1**	■		□	
**GATA3**					**RUNX1**		■		□
**Gfi**					**RUSH1-alfa**				
**HAND1-TCF3**		■		□	**RXRA-VDR**				
**HLF**					**RXR-VDR**				
**HNF1A**					**Sox17**		■	□	
**HNF4**					**Sox5**				□
**Hox11-CTF1**					**SOX9**				
**IRF1**		■	□	□	**SP1**	■		□	
**IRF2**					**SPI1**				
**Klf4**	■		□		**SPIB**		■		□
**Lhx3**		■	□	□	**Spz1**				
**MafB**	■		□		**SRF**				
**MAX**					**SRY**				
**MEF2A**					**Staf**				□
**MIZF**					**STAT1**				
**Myb**					**T**				
**MYC-MAX**					**TAL1-TCF3**				
**Mycn**					**TBP**				
**Myf**			□		**TCF1**				
**MZF1_1-4**	■		□		**TEAD**		■		□
**MZF1_5-13**	■		□		**TFAP2A**			□	
**NFIL3**					**TP53**		■	□	□
**NF-kappaB**			□		**USF1**				
**NFKB1**			□		**YY1**				
**NF-Y**					**ZEB1**			□	
					**ZNF42_1-4**	■		□	

All 101 JASPAR PWMs that were mapped onto the zebra finch genome assembly are listed. Blocks in the row denote the presence of the PWM either in the 5'-focused region (filled in blocks) or across the entire gene territory (open blocks) for StAR and TSPO.

**Table 3:** Qualitative listing of PWM predictions compiled from CYP19 and HSD17B4 genes.

	CYP19	HSD17B4	CYP19	HSD17B4		CYP19	HSD17B4	CYP19	HSD17B4
**Ar**					**NFYA**		■	□	□
**Arnt**		■		□	**NHLH1**		■		□
**Arnt-Ahr**		■	□	□	**Nkx2-5**			□	□
**Bapx1**			□	□	**NKX3-1**			□	
**cEBP**			□	□	**Nobox**				□
**CREB1**			□	□	**NR1H2-RXR**	■		□	□
**Ddit3-Cebpa**	■		□	□	**NR2F1**				□
**E2F1**		■		□	**NR3C1**			□	□
**ELF5**				□	**Pax2**				□
**ELK1**		■		□	**Pax4**			□	□
**En1**			□	□	**Pax5**		■		□
**ESR1**			□	□	**Pax6**			□	□
**ETS1**		■	□	□	**Pbx**			□	□
**Evi1**			□	□	**Pdx1**			□	□
**Fos**				□	**PPARG**			□	□
**Foxa2**				□	**PPARG-RXRA**	■		□	□
**FOXC1**				□	**Prrx2**			□	□
**Foxd3**					**REL**			□	□
**FOXF2**	■		□	□	**RELA**			□	
**FOXI1**			□	□	**REST**	■		□	
**FOXL1**			□	□	**Roaz**		■	□	□
**Foxq1**			□	□	**RORA_1**		■	□	□
**GABPA**		■	□	□	**RORA_2**		■		□
**Gata1**			□	□	**RORA1**		■		
**GATA2**			□	□	**RREB1**				□
**GATA3**			□	□	**RUNX1**	■		□	□
**Gfi**			□	□	**RUSH1-alfa**				□
**HAND1-TCF3**			□	□	**RXRA-VDR**	■		□	□
**HLF**				□	**RXR-VDR**	■		□	□
**HNF1A**				□	**Sox17**				□
**HNF4**		■			**Sox5**			□	
**Hox11-CTF1**				□	**SOX9**			□	□
**IRF1**	■		□	□	**SP1**		■		□
**IRF2**				□	**SPI1**				□
**Klf4**				□	**SPIB**		■		□
**Lhx3**	■		□	□	**Spz1**	■		□	□
**MafB**		■		□	**SRF**	■		□	□
**MAX**					**SRY**			□	□
**MEF2A**			□		**Staf**				
**MIZF**		■			**STAT1**			□	□
**Myb**		■	□	□	**T**			□	□
**MYC-MAX**					**TAL1-TCF3**			□	□
**Mycn**		■			**TBP**		■	□	□
**Myf**				□	**TCF1**				□
**MZF1_1-4**		■		□	**TEAD**				
**MZF1_5-13**		■		□	**TFAP2A**		■		□
**NFIL3**			□	□	**TP53**				
**NF-kappaB**		■			**USF1**	■	■	□	
**NFKB1**					**YY1**	■		□	□
**NF-Y**		■	□	□	**ZEB1**				
					**ZNF42_1-4**		■		□

All 101 JASPAR predictions mapped onto the zebra finch genome assembly are listed. Blocks in the row denote the presence of the PWM either in the 5'-focused region (filled in blocks) or across the entire gene territory (open blocks) for CYP19 and HSD17B4.

### Neural expression of TSPO

The finding that StAR and TSPO shared few PWM sites suggested that these genes may have largely independent transcriptional regulatory mechanisms, which could result in distinct patterns of expression. Non-overlapping expression distributions would be inconsistent with recent models that propose that StAR and TSPO work together in a complex that is required for transporting cholesterol across the mitochondrial membranes for the initiation of steroidogenesis [26]. TSPO is one of the few components of the estradiol-synthetic pathway not yet examined in the zebra finch brain. We therefore utilized information from cDNA and microarray resources developed alongside the genome assembly to confirm that TSPO was expressed in the zebra finch brain, test whether or not it co-localized to brain areas that expressed StAR, and validate results from a zebra finch brain microarray that indicated that its mRNA was rapidly regulated after experience [56,57].

**Figure 5:**
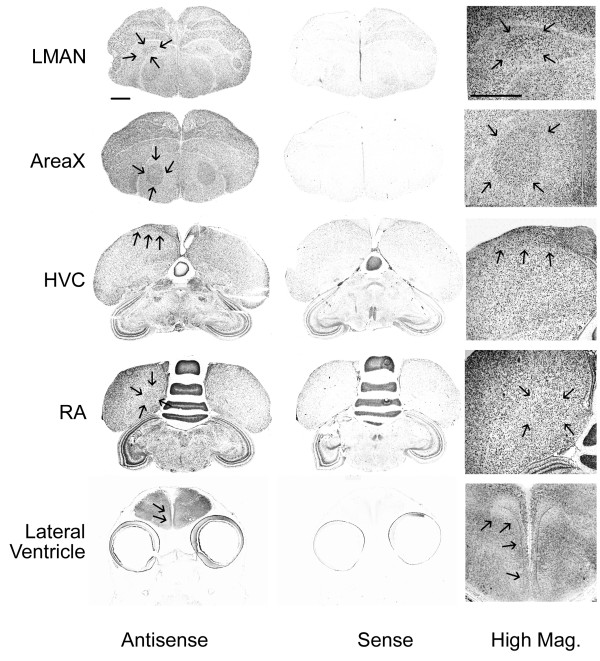
**TSPO in situ hybridization in adult song system and along P1 lateral ventricles**. In situ hybridization with antisense-configured riboprobes showed that TSPO is expressed within all four major song nuclei - LMAN, Area X, HVC, and RA - in adult male brain. Antisense-configured riboprobes do not label the cells surrounding the lateral ventricle in P1 brains. Song nuclei and region of the lateral ventricle are identified with arrows. Hybridization of adjacent brain sections with negative control sense-configured riboprobes shows very low levels of label, suggesting specific labeling with antisense probes. High magnification images of each region of interest show cellular labeling. Scale bars for whole brain images = 1 mm; scale bar for high magnification images = 500 μm.

Several TSPO ESTs were identified in the zebra finch ESTIMA collection of brain cDNAs indicating that it was transcribed in the brain [57]. We used one of these clones to perform in situ hybridization to test if TSPO expression was neuroanatomically distributed within two functional regions known to express StAR and other steroidogenic genes: the nuclei of the song control system in adult males, and the cells along the proliferative zone of lateral ventricle in posthatch day 1 (P1) birds. In situ hybridization showed that TSPO is expressed in all four major song nuclei in adult male birds (Figure 5). The intensity of labeling within these nuclei is not noticeably above that in the surrounding brain, and its hybridization distribution is widespread. In P1 birds, TSPO hybridization occurred at low levels throughout the brain but was strikingly absent from the cell-dense region surrounding the lateral ventricle (Figure 5). Hybridization of adjacent sections with sense riboprobe did not show any labeling in adult or P1 brains, suggesting that the observed hybridization patterns are specific for TSPO.

**Figure 6:**
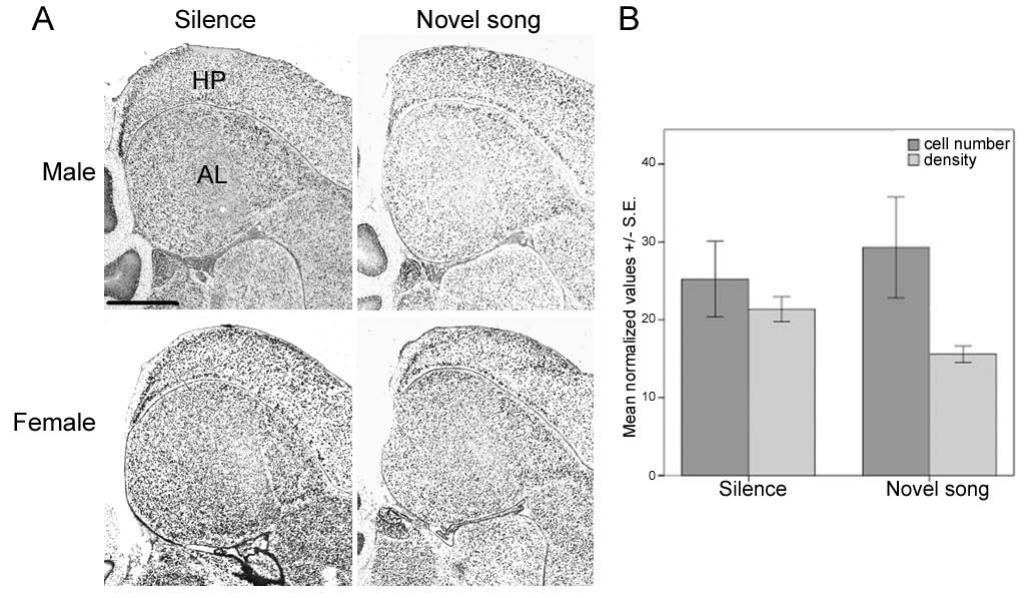
**TSPO in situ hybridization in the adult AL after song playback experience**. A) Representative images of AL (teardrop-shaped brain area in center of images) from males and females that heard either Silence or Novel song. B) The intensity of TSPO labeling showed a non-significant trend (p = 0.057) towards a decrease in birds that heard novel song. AL = auditory forebrain lobule, HP = hippocampus. Scale bar = 500 μm.

We also measured TSPO and StAR expression in the auditory forebrain lobule (AL; [58]), a brain area required for song processing and learning [56,59-61]. Adult birds were either placed in silence or played 30 minutes of novel zebra finch song, and their brains were processed for in situ hybridization. In the AL, there was a strong but non-significant trend of song condition on the intensity of TSPO labeling in cells (p = 0.057) because labeling was less intense in the Novel song condition compared to the Silent condition (Figure 6). Hearing song had no effect on the total number of TSPO-labeled cells in AL (p = 0.202). In one set of sections, we detected a main effect of sex on the number of TSPO-labeled cells (p = 0.031), with males having more cells than females in the AL, but this effect was not confirmed with a second, independent set of sections from other birds analyzed the same way. Sex had no effect on the average intensity of cell labeling (p = 0.190), and there was no significant interaction between sex and song condition with respect to the number of TSPO-labeled cells (p = 0.387) or the intensity of cell labeling (p = 0.173). We found no significant effects of song condition (cell number p = 0.341; labeling intensity p = 0.538), sex (cell number p = 0.189; labeling intensity p = 0.099), or the song condition by sex interaction (cell number p = 0.564; labeling intensity p = 0.421) on the level of StAR hybridization in AL.

## Discussion

Steroids exert powerful effects on many physiological systems across animals. In the zebra finch, they are particularly relevant because they can be synthesized within the brain and may have a special role in the sexual differentiation of the developing song system and as rapid signaling molecules in the adult song system. We took advantage of the newly-released zebra finch genome assembly to investigate the structures of the steroid-related genes. We assess the diversity of their gene families and putative transcriptional regulatory elements, and contribute additional neuroanatomical expression data regarding the potential for regulated neurosteroidogenesis.

We first used homology searches to identify twenty steroid-related genes in the zebra finch genome assembly. After comparing zebra finch genome sequence to gene sequences from other species and zebra finch cDNA information, we determined that many of the genes were incomplete in the assembly, likely due to technical assembly gaps. As expected, cross-species analysis of the gene sequences did generally show high homology to genes in other species, and the preservation of major enzyme active sites and overall domain structure of the receptors.

Over half of the twenty genes we analyzed from the genome assembly belong to one of two enzyme families, HSD3B and HSD17B. We were, however, unable to identify the full complement of genes in either of these families as described in mammals. It is possible that zebra finches do have all of the HSD3B and HSD17B genes that mammals have and that their absence in the assembly simply reflects incomplete genome sequencing. But based on evolutionary theories of the expansion of these gene families and the phylogenetic analysis of their predicted protein sequences performed here, it is likely that there is a biological, not technical, basis for this difference in gene family sizes. For example, up to 7 different HSD3B genes have been discovered in other animals, each encoding a distinct enzyme. In mammals, genes for HSD3B1-6 encode steroid-metabolizing enzymes that are believed to have arisen from duplication events, in part because they are localized to the same chromosome [35-37]. HSD3B7 is considered primarily a bile-synthesizing enzyme and may have evolved independently from the other HSD3B proteins, as it is localized to a different chromosome as shown here and in mammals [38]. Previously in the zebra finch, a cDNA for HSD3B1 had been cloned, and there was evidence for multiple HSD3B-like transcripts in both the gonads and the brain [3]. The zebra finch HSD3B1 gene was mapped to chromosome 1, but our homology searches were unable to identify any other HSD3B genes on this chromosome. Efforts to identify these genes in the chicken genome assembly were also unsuccessful. Our current findings therefore suggest that the HSD3B gene expansion that occurred in mammals did not occur in the avian lineage.

Similarly, we could identify only nine of the fourteen mammalian HSD17B genes in the zebra finch genome assembly. The enzymes of the HSD17B family catalyze reactions with different specificities and affinities for androgens and estrogens, as well as fatty acids, retinoids, and cholesterol [30,39,62]. Two HSD17B genes, HSD17B3 and HSD17B4, were mapped to the Z sex chromosome; other studies confirmed the Z chromosome localization of HSD17B4 [40] London, Itoh, Lance, Ekanayake, Oyama, Arnold, Schlinger: Neural expression and post-transcriptional dosage compensation of the steroid metabolic enzyme 17β-HSD type 4: submitted. This indicates the potential for different levels of expression or activity of these enzymes in males and females, as genes on the zebra finch Z chromosome undergo incomplete dosage compensation [18,41]. This leads to a male-bias in expression of Z-linked genes that may be relevant to the organization or function of the steroid-sensitive sexually dimorphic song system [18,41-45] London, Itoh, Lance, Ekanayake, Oyama, Arnold, Schlinger: Neural expression and post-transcriptional dosage compensation of the steroid metabolic enzyme 17β-HSD type 4: submitted. Interestingly, the zebra finch HSD17B3 gene may be distinct from HSD17B3 in other species because we were unable to build a complete gene model based on cross-species sequence homology. The HSD17B4 gene and protein was further investigated elsewhere to examine the potential for sex differences in neural expression and activity [45] London, Itoh, Lance, Ekanayake, Oyama, Arnold, Schlinger: Neural expression and post-transcriptional dosage compensation of the steroid metabolic enzyme 17β-HSD type 4: submitted. Our inability to identify the five other HSD17B genes described in mammals may simply reflect low sequence homology across species [30,39,62]. Alternatively, it may be that some of the HSD17B types have evolved in mammals but not birds. Most HSD17B genes are thought to have evolved independently [30,39,62,63]; some of the missing enzymes display some functional redundancies with HSD17B enzymes we could identify in the zebra finch. Perhaps then the complement of HSD17B enzymes in zebra finches can perform all of the necessary reactions represented in the fourteen mammalian enzymes [32,39,63-66].

Phylogenetic analysis of the HSD3B and HSD17B predicted protein sequences suggested that the HSD17B zebra finch and mammalian isoforms are similar to each other, but that the bird HSD3B7 enzyme may have a slightly different function than that in mammals. For example, in the HSD17B family, HSD17B types 4 and 7 were placed on their own branches of the phylogenetic tree. This is consistent with the fact that the HSD17B4 enzyme has a unique set of catalytic structures and that HSD17B7 may have sequence structure that is optimized for cholesterol synthesis rather than the steroid or fatty acid metabolism more commonly performed by the other HSD17B enzymes [32,67-70]. Further, HSD17B types 3 and 12 were closely associated on the tree, as were types 2 and 6. HSD17B3 and HSD17B12 show higher sequence similarity in humans, too, and it has been previously postulated that HSD17B2 and HSD17B6 are derived from a common ancestral invertebrate enzyme [71-74]. On the other hand, phylogenetic modeling of the HSD3B predicted amino acid sequences suggested that the chicken and zebra finch HSD3B type 7 proteins are distinct from those in other species. If all of the steroidogenic conversions performed by the isozymes in mammals are necessary in birds, too, it may be that one of the two avian HSD3B enzymes can catalyze several reactions that are divided amongst HSD3B types 2-6 in other animals.

The extent to which different components in the steroidogenic pathway are co-expressed can influence the local steroid concentration and mixture, and the pattern of receptor expression can directly affect the signaling efficacy of those steroids. We therefore examined, both quantitatively and qualitatively, the profile of PWMs in these genes. Using Ensembl models and the genome-wide mapping of PWM sites, we identified two transcription factors, NR1H2-RXR and Nobox, that were overrepresented either in the entire set of genes or within the nuclear receptor genes, respectively. Both NR1H2-RXR and Nobox have known relationships with steroids and may therefore be relevant to steroid synthesis and signaling in the zebra finch [75,76]. A comparison of predicted PWM sites in CYP19 and HSD17B4, genes that code for estradiol synthesizing and metabolizing enzymes, suggested that many regulatory elements are common between the two genes and indeed, some transcription factors have been identified that regulate expression of both genes in other systems such as steroidogenic cell lines [24,77-79]. Although accurately predicting active transcription factor binding sites is complicated and certainly not definitive, this analysis suggests some transcription factors to target in future experiments that investigate mechanisms of steroid regulation in the zebra finch.

The zebra finch brain produces estradiol; there is ample evidence from a variety of methods that demonstrate that steroidogenic enzymes are present and active within the brain, and that brain slices, which cannot receive steroid precursors from the periphery, synthesize estradiol de novo [1,3-15,18,19,45] London, Itoh, Lance, Ekanayake, Oyama, Arnold, Schlinger: Neural expression and post-transcriptional dosage compensation of the steroid metabolic enzyme 17β-HSD type 4: submitted. However, the precise pathways and locations of steroid synthesis in the brain are still uncertain. According to one recent proposal based on in vitro experiments, the StAR and TSPO proteins may work together in a complex that controls the transport of cholesterol from the outer mitochondrial membrane to the inner mitochondrial membrane, effectively controlling the initiation of steroidogenesis [26]. However, we observed minimal overlap in PWM sites associated with these two genes, and only partial neuroanatomical colocalization of the two mRNAs when our TSPO results here were compared with previous studies of StAR brain expression [3,19]. Notably, only one of the two transcripts is found in two brain regions relevant to the development and function of the steroid-sensitive song system: Area X of the adult male song circuit and the region surrounding the lateral ventricle in P1 birds. These results suggest either that StAR and TSPO coexpression are not absolutely necessary for steroidogenesis in brain tissue, or that neurosteroid synthesis is occurring at sites other than the current foci of research. As further evidence that transcription of these two genes is controlled differently, TSPO showed a strong trend towards regulated transcription in the AL after birds experienced song playbacks, consistent with a previous report, but levels of StAR mRNA were unchanged across the same conditions [56]. The current findings emphasize the importance of investigating these genes in the tissue where they function, and indicate that more experiments that delve into the cholesterol transport mechanisms for steroidogenesis in the brain are required.

## Conclusions

We took advantage of the zebra finch genome assembly to integrate genomic and neural investigation of the enzymes and receptors of the estradiol-synthetic pathway. While mechanisms of steroid synthesis and action are largely conserved across phylogeny, the zebra finch and other birds may have evolved several unique features. Notably, genomic and molecular analysis of two major cholesterol transport proteins suggests that the regulation of steroidogenic initiation may be even more complex than previously. believed.

## Methods

All procedures that involve animals were approved by the University of Illinois, Urbana-Champaign Institutional Animal Care and Use Committee.

### Identification of steroidogenic genes

Whenever possible (StAR, TSPO, CYP11A1, HSD3B1, CYP17, CYP19, HSD17B4, ERα, ERβ, AR, PR), we used existing zebra finch cDNA sequence to do homology searches of the zebra finch genome trace archive http://www.ncbi.nlm.nih.gov/genome/seq/BlastGen/BlastGen.cgi?taxid=59729 and the genome assembly http://genome.ucsc.edu. When no zebra finch sequence was available, we used homology searches of the assembly to identify genes with available chicken sequences (HSD3B7, HSD17B2), or human sequence if a chicken gene model was not available (HSD17B1, HSD17B3, HSD17B6, HSD17B7, HSD17B10, HSD17B11, HSD17B12). We also used sequence from specific conserved functional domains for homology searches to attempt to identify enzyme genes. All genomic sequences were corrected using cDNA and protein information from zebra finch, chicken, mouse, and human in Apollo Genome Annotation Curation Tool [80]. We used zpicture http://zpicture.dcode.org/ to visualize conservation of gene structures and completeness of genomic sequences.

### PCR with genomic DNA

We used PCR with genomic DNA to validate one of the Ensembl gene models for a gene that had not been cloned previously. DNA was extracted from zebra finch tissue samples and purified with DNeasy kit using manufacture's instructions (Qiagen, Valencia, CA). PCR was performed with HotStarTaq (Qiagen) for 55 cycles, products were gel extracted (Gel Extraction, Qiagen), and ligated into PCRScript Amp plamsid (Stratagene, La Jolla, CA). Clones were sequenced on both strands and their identities were confirmed by BLAST homology searches. We then aligned the clone sequence to that of the genome assembly to test for sequence matches.

### Phylogenetic analysis

Predicted amino acid sequences for zebra finch genes were aligned with a combination of human, mouse, chicken, platypus, and zebrafish in MAFFT [81,82], and edited when necessary in BioEdit http://www.mbio.ncsu.edu/BioEdit/bioedit.html. Unrooted neighbor-joining phylogenic trees of three gene families, HSD3B, HSD17B, and the nuclear receptors were constructed with MEGA4 [83,84]. Bootstrap support was performed with 1000 replications; consensus trees are shown.

### Distribution of transcription factor binding motifs

We performed a statistical test for overrepresented PWM sites within the territories of the Ensembl gene models using the JASPAR http://jaspar.cgb.ki.se/ set of non-redundant and curated PWMs [54]. This tested if any of the JASPAR PWM sites were more abundant in the steroid-related gene models compared to their distribution across the whole genome assembly. We investigated several sets of genes: all of the 18 genes from our whole set of 20 that had Ensembl models, just StAR and TSPO, just CYP19 and HSD17B4, all of the predicted components of the estradiol-synthetic pathway (StAR, TSPO, CYP11A1, CYP17, HSD3B1, HSD17B1, HSD17B2, HSD17B4, CYP19), and the four nuclear receptors (ERα, ERβ, AR, and PR).

We also utilized the JASPAR PWM predictions across the whole genome assembly to perform a purely qualitative description of two pairs of functionally related genes that had complete coding regions and few gaps in the assembly: StAR and TSPO, and CYP19 and HSD17B4 [54]. We performed this inventory on two regions of the gene. One, focused on the putative 5' proximal regulatory region, captured the PWMs that were 5 kb upstream and 2 kb downstream of the 5'-most exon; the second, designed to identify regulatory elements scattered across the entire gene region, captured the PWMs within the boundaries of the entire gene plus those contained within 5 kb both 5' and 3' to the predicted gene model. This whole gene analysis was done with two caveats. To be conservative about what we included in this analysis, we did not extend the PWM characterization across an assembly gap unless there was cDNA evidence that the gene indeed spanned the gap, nor did we include PWMs that were within 5 kb of the gene of interest if an adjacent gene was predicted to fall within that boundary. In those cases, we only describe PWM that are non-overlapping with the adjacent gene.

### In situ hybridization

The region along the lateral ventricles at P1 and the song control nuclei of adult males were previously identified to be brain areas that express genes in the estradiol-synthetic pathway [3,9,19,85]. Here, we performed in situ hybridization for TSPO on P1 (n = 3 males, n = 3 females) and adult male (n = 3) brains to determine whether or not it was also expressed in these two regions. P1 birds were removed from their nests in a breeding aviary and adult males were removed from single-sex holding aviaries located in a room that housed both males and females. Within 3 minutes of removal from their housing environment, birds were sacrificed and brains were flash frozen and sectioned to 20 μm in the coronal plane. Sex of the P1 birds was confirmed by visual inspection of the gonads. The TSPO DIG-labeled riboprobe was in vitro transcribed using a zebra finch brain EST from the Songbird Neurogenomic Initiative's ESTIMA collection (GenBank Accession number DV952129) as the template [57]. This EST is predicted to contain the entire open reading from of the TSPO transcript and is highly specific for TSPO (e score = 1e-180). Sense riboprobes were also synthesized as negative hybridization controls. For every slide hybridized with the antisense probe, an adjacent slide was hybridized with the sense riboprobe for control. Labeling patterns on brain sections hybridized with antisense or sense probes were compared to evaluate if specific hybridization occurred along the lateral ventricles and in major song nuclei.

In situ hybridization was performed for all slides as follows [86]. The tissue was postfixed for 15 minutes in 4% paraformaldehyde (pH 7.4), then washed 4 × 5 minutes in 0.02 M KPBS (pH 7.4). The slides were equilibrated in TEA and treated with 0.25% acetic anhydride in TEA for 10 minutes, then rinsed in 2× SSC. Finally, the sections were dehydrated through increasing concentrations of ethanol. Hybridization with 500 ng of riboprobe (hybridization solution: 50% formamide, 2× SSPE [pH 7.4], 2 mg/ml tRNA, 1 mg/ml bovine serum albumin, 300 ng/ml polyadenylic acid, 0.1 M dithiothreitol) proceeded for 3 hours at 65°C. After hybridization, slides were rinsed in 2× SCC at room temperature, then washed in 50% formamide/1× SSC for 10 minutes at 65°C with 2 × 20 minute final high stringency washes in 0.1 X SSC, all at 65°C. Slides were then placed in blocking buffer (1% blocking reagent (Roche Applied Science, Indianapolis, IN) in buffer A (100 mM Tris [pH 7.5], 150 mM NaCl, and 0.05% Triton X-100) at 4°C overnight. The next day, slides were rinsed in buffer A and incubated for 3 hours at room temperature with alkaline phosphatase conjugated anti-DIG antibody (Roche Applied Science) diluted 1:5000 in blocking buffer. Slides were washed 4 × 5 minutes in buffer A, then in buffer B (100 mM Tris [pH 9.5], 100 mM NaCl, and 50 mM MgCl_2_) for 10 min. Color detection was performed with BCIP/NBT alkaline phosphatate substrate (Sigma-Aldrich, St. Louis, MO).

A previous study reported that TSPO mRNA showed a relatively rapid (within 30 minutes) decrease in levels in the AL after an adult male bird heard novel conspecific song [56]. Therefore, we also used in situ hybridization to confirm this finding and extend the investigation of TSPO expression in the AL to females. We also performed in situ hybridization for StAR using a clone previously described to compare TSPO and StAR mRNA changes after song playback experience [3]. We used adult male and adult female birds (n = 3 each sex) and exposed them to a standard song playback paradigm. Birds, who had been group-housed in single sex aviares in a room that housed both males and females, were removed from this communal setting and placed individually in a sound isolation chamber overnight. The next morning, birds were either played a zebra finch song unfamiliar to the bird for 30 minutes (one song bout every 10 seconds, for a total of 180 bouts; "Novel song") or left in silence ("Silence"), and sacrificed immediately after song playback ceased (or, for the Silence birds, within 30 minutes of the Novel song birds). To ensure that the song used for playback was unfamiliar to the experimental birds, we used a song recorded more than 10 years ago from a bird that was no longer present in the aviary population. Brains were extracted, flash frozen, and sectioned in the sagittal plane to 12 μm. In situ hybridization was performed on three AL sections ~340 μm apart to represent the medial-lateral extent of the auditory forebrain for each bird. Sections were hybridized with the TSPO and StAR riboprobes under the conditions described above [86].

Low magnification images of the adult male coronal brain sections were captured with a Nikon slide scanner (Super Coolscan 8000ED; Nikon Inc., Melville, NY). Images of P1 brains, individual song nuclei, and AL-containing brain sections were digitally captured on an AxioImager A1 Microscope (Carl Zeiss Microimaging, Thornwood, NJ) with a CCD camera (Microfire; Optronics, Goleta, CA). Representative in situ hybridization images shown were modified for contrast to highlight levels and areas of gene expression; modifications were performed equally for all images of a set.

The number and intensity of labeled cells in the AL was quantified within the entire AL and the adjacent non-auditory hippocampus (HP) for control purposes using ImageProPlus 4.5.1 (MediaCybernetics; Bethesda, MD) [87]. To remove potential differences in AL staining pattern across slides or from non-specific background staining, each AL value was normalized to the HP value on the same section. We then calculated a "total AL" value by summing the normalized values for all the three AL sections obtained in each bird. Normalized AL values were analyzed with two-way ANOVA (SPSS, Chicago, IL; α = 0.05) to test for effects of sex, song condition, and the sex by song condition interaction.

## Authors' contributions

SEL designed, performed, and interpreted the experiments and analyses, and wrote the manuscript. DFC participated in the design of experiments and analyses and contributed to manuscript writing. Both authors read and approved this manuscript.

## Supplementary Material

Additional file 1 **Figure S1 - Conservation of gene structure for nineteen zebra finch genes, represented by zpictures**. Zebra finch sequences from the genomic assembly, and when available, full length cDNA clone sequences, aligned to annotated genes to show evolutionarily conserved regions. When annotated, chicken genes were used as the base gene, otherwise the human gene was used. Percent sequence homology of regions above 50% similarity is depicted by the height of the colored bars. Direction of arrows at the top of each gene's alignment indicates the 5' to 3'direction of the base sequence. Click here for file

Additional file 2 **Figure S2 - Gene models showing alternative transcripts**. Images of gene models corrected in Apollo Genome Annotation Curation Tool, showing predicted alternative transcripts based on evidence in other species. Position on the chromosome is depicted by the scale across the bottom of each gene. Dark blue portion denotes coding sequence, light blue denotes untranslated regions, green and red stripes represent position of translational start and stop codons, respectively. Click here for file

Additional file 3 **Figure S3 - Alignment of the HSD17B1 sequence from the zebra finch assembly and from the clone obtained from PCR amplification from zebra finch genomic DNA**. Sequence alignment of the genomic HSD17B1 gene from the assembly aligned in ClustalX to the clone amplified directly from zebra finch DNA, validating a portion of this sequence. Click here for file

Additional file 4 **Figure S4 -Unrooted phylogenetic tree of nuclear receptor predicted protein sequences**. Unrooted phylogenetic trees of the four nuclear receptor types examined model the zebra finch ER receptor subtypes as more similar to each other than AR and PR, which are more closely related to each other. Cross-species positioning within each receptor type show evolutionary changes between birds and mammals. Bootstrap support values are at branch points. Scale bar denotes substitution rate. zf = zebra finch, ch = chicken, h = human, m = mouse, dan = zebrafish, platy = platypus, iso = isoform. Click here for file
