# Supplementary figures and images for: Genomic and neural analysis of the estradiol-synthetic pathway in the zebra finch
Source: BMC Neurosci. 2010 Apr 1;11:46. doi: 10.1186/1471-2202-11-46 (PMC2865489; doi:10.1186/1471-2202-11-46)

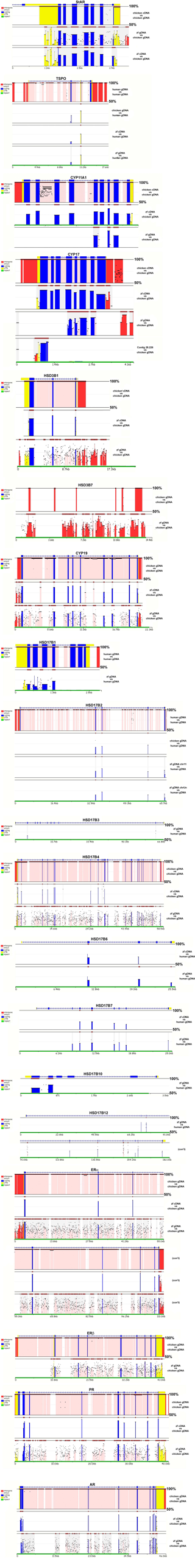

Supplement: Additional file 1 — Figure S1 - Conservation of gene structure for nineteen zebra finch genes, represented by zpictures. Zebra finch sequences from the genomic assembly, and when available, full length cDNA clone sequences, aligned to annotated genes to show evolutionarily conserved regions. When annotated, chicken genes were used as the base gene, otherwise the human gene was used. Percent sequence homology of regions above 50% similarity is depicted by the height of the colored bars. Direction of arrows at the top of each gene's alignment indicates the 5' to 3'direction of the base sequence. [file 1471-2202-11-46-S1.JPEG]

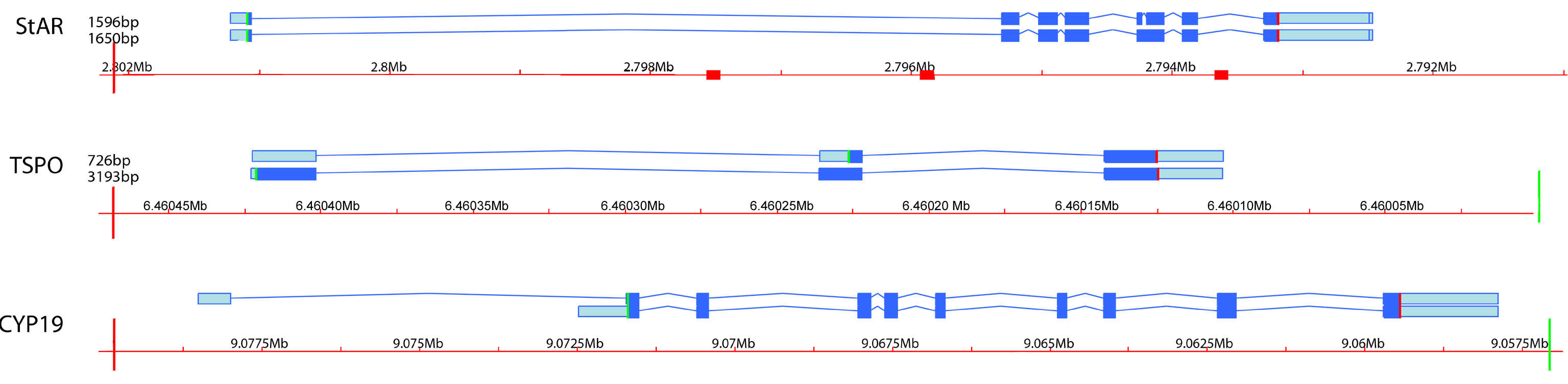

Supplement: Additional file 2 — Figure S2 - Gene models showing alternative transcripts. Images of gene models corrected in Apollo Genome Annotation Curation Tool, showing predicted alternative transcripts based on evidence in other species. Position on the chromosome is depicted by the scale across the bottom of each gene. Dark blue portion denotes coding sequence, light blue denotes untranslated regions, green and red stripes represent position of translational start and stop codons, respectively. [file 1471-2202-11-46-S2.JPEG]

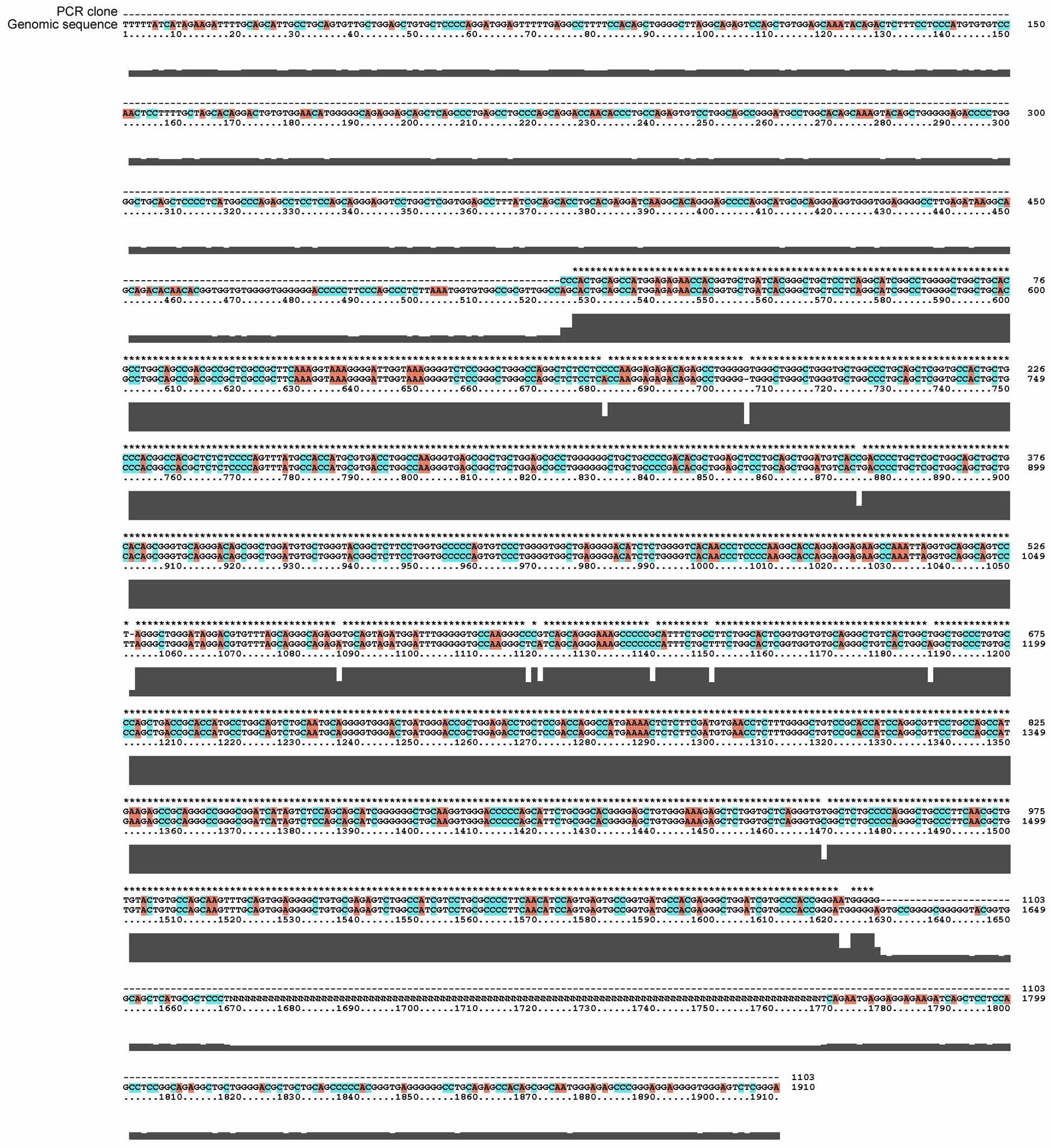

Supplement: Additional file 3 — Figure S3 - Alignment of the HSD17B1 sequence from the zebra finch assembly and from the clone obtained from PCR amplification from zebra finch genomic DNA. Sequence alignment of the genomic HSD17B1 gene from the assembly aligned in ClustalX to the clone amplified directly from zebra finch DNA, validating a portion of this sequence. [file 1471-2202-11-46-S3.JPEG]

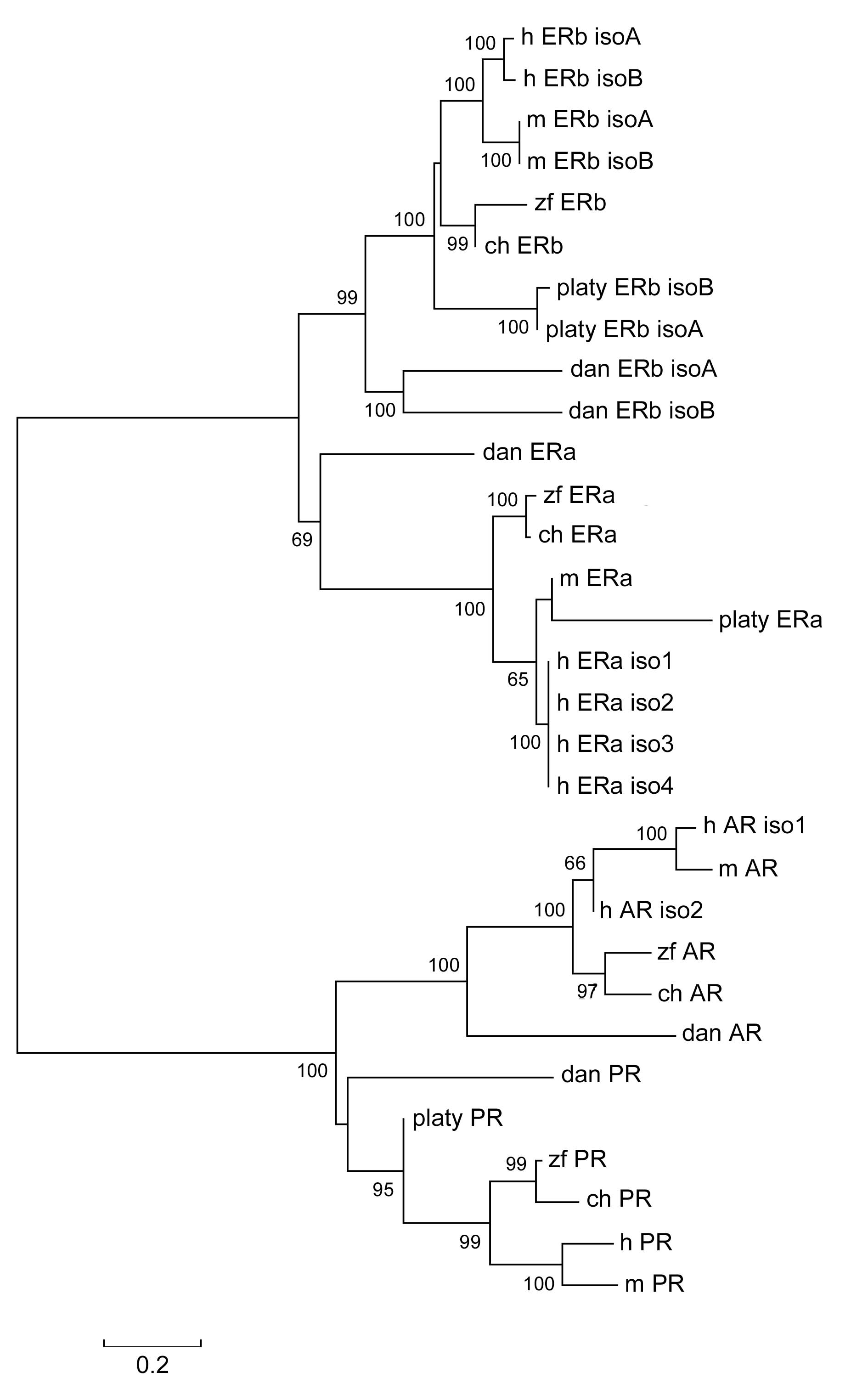

Supplement: Additional file 4 — Figure S4 -Unrooted phylogenetic tree of nuclear receptor predicted protein sequences. Unrooted phylogenetic trees of the four nuclear receptor types examined model the zebra finch ER receptor subtypes as more similar to each other than AR and PR, which are more closely related to each other. Cross-species positioning within each receptor type show evolutionary changes between birds and mammals. Bootstrap support values are at branch points. Scale bar denotes substitution rate. zf = zebra finch, ch = chicken, h = human, m = mouse, dan = zebrafish, platy = platypus, iso = isoform. [file 1471-2202-11-46-S4.JPEG]
